# The Purins1Read before the Buffalo Academy of Medicine, October 11, 1910.

**Published:** 1910-11

**Authors:** Henry Reed Hopkins

**Affiliations:** Buffalo, N.Y.; Ninety-seventh President of the Medical Society of the State of New York, (1903); Honorary Life Member of the Medical Society of the County of Erie; 433 Franklin Street


### BUFFALO MEDICAL JOURNAL

VOL. LXVI. NOVEMBER, 1910. No. 4

### ORIGINAL COMMUNICATIONS

### The Purins[1]

BY HENRY REED HOPKINS, M.D.
Buffalo, N. Y.
Ninety-seventh President of the Medical Society of the State of New York, (1903);
Honorary Life Member of the Medical Society of the County of Erie.

OUR knowledge of the purin bodies, like many matters of human achievement, presents for our consideration quite distinctive phases or periods, both artistic and scientific. The period of faith, of fancy, of hope, of belief, of conviction, the period peculiar to the art of medicine, began in the early days of our knowledge of vital chemistry and will prevail for many generations to come. The period characterised by the use of more exact methods, the scientific period, dates from our acquaintance with the chemistry of urea which we owe to Rouelle, Fourcroy and Vanquelin, 1773 to 1799, and with greater particularity from the discovery by Scheele in 1776 of the chemical composition and relations of uric acid. It may be said with historical propriety that the dawn of the scientific period of our knowledge of this subject dates from 1773, but the rising sun of the matter is the work of Emil Fischer, his discovery of the hypothetical chemical molecule $C_5N_4$ in 1899—the nucleus from which uric acid and a large number of related chemical substances are formed, which hypothetical chemical molecule Fischer named Purin, the characteristic component of the purin bases and purin bodies, these said purins being intimately concerned and related to the anabolism and the catabolism of nitrogen, that most interesting constituent of organised bodies of plants and animals.

Our thoughts of the purins may properly be directed in the interest of accuracy and greater efficiency along the following lines:

1. The Purin diathesis and purin diseases.
2. The Purins and heredity.
3. The Purins endogenous and exogenous.
4. The Purin, rich, poor and free foods.

---

1. Read before the Buffalo Academy of Medicine, October 11, 1910.

5. The Purins and old age.
6. The Purins in modern dietotherapy.
7. Conclusions.

### THE PURIN DIATHESIS AND THE PURIN DISEASES.

It is with distinct timidity and hesitation that the speaker comes before you with a term of such dubious parentage and record as the word diathesis. You will therefore bear with me while making the disclaimer to the effect, that much which has been claimed for the word represents enthusiasm without knowledge, that the conclusions of the men from our laboratories as to the misuse and the overuse of the word are valid, that the constant growth in the domain of etiology of the role of the various pathogenic organisms, is at the expense of the territory supposed to belong to diathesis and is of the nature of real progress. These and other like contentions should be conceded without question; and yet the fact remains, that many of our wise medical men know that men are born with a pathologic, germinal, protoplasmic, variation from the normal, resulting in a general predisposition to a given disease as gout, diabetes, tuberculosis or hemophilia; or to a more or less abnormal condition of the nervous system known as a neuropathic state, and to this pathologic predisposition they give the name—diathesis.

Our interest in the question of diathesis comes from the prominent role played by the purins in the production of gout, or some of the many manifestations of diseased function known as gouty, or goutiness. The pertinent facts seem to be like this: For centuries it has been observed that the overuse of certain foods was in the gouty followed by an attack of gout. These foods are now known to be rich in purins. The case seems to be proved that men suffering with the gouty, or as we may now say the "Purin Diathesis," have less than the normal ability to metabolise purin bearing foods; that when men use such foods this defective catabolism is shown by the presence in the excretions of unusual amounts of purin bodies, and experiment has proved that these purin bodies as compared with urea are distinctly poisonous.

My observations of the sick and my study of the literature of the subject, leaves no doubt in my mind as to the enormously important practical fact, that many of our patients suffer with the purin diathesis and it is only possible for us to protect and preserve them from the disabling results of the purin diseases, by an intelligent and persistent regulation in their diet, of the purin bearing foods.

This subject would merit your most careful consideration if the above premises and conclusions were true and applicable only to our distinctly arthritic patients. In my judgment the cases of gout and rheumatism, the arthritics, constitute but a small proportion of the purin diseases, that this group properly includes an enormous number of cases of complainers in which the complaints embrace or indicate functional disturbances of most of the viscera, organs and tissues. In my experience a diagnosis of a purin disease is made upon three factors:

First, a clinical history of some functional disturbance or impairment having usually the three distinguishing characteristics of persistency, variability and intermittence.

Second, the history of persistent use or overuse of purin rich foods, frequently with the history of the possible or probable possession of the purin diathesis.

Third, evidence in the urine of faulty metabolism, suboxidation, low urea and high purin bodies, uric acid, xanthins, guanins, adenins, being the more frequent.

This enormous group of cases, so large that the medical enthusiast might make them cover the whole clinical heavens, exists as the result of the faulty catabolism of our nitrogen bearing foods and the consequent autointoxication, concerning which we were first taught by Bouchard and subsequently by von Noorden. Of the catabolic products of this suboxidising catabolism the purin bodies have the chief role.

But I hear some of you protest, " Don't talk to us about Bouchard and his seven toxins; he and his work have been discredited again and again." To which protest it may be remarked that undoubtedly Bouchard like many a pioneer was guilty of enthusiasm, and some of his methods and conclusions have not been and may never be confirmed by competent observers. Nevertheless, my conviction is positive that in the estimation of the more recent students,—German, English and American,—the main contentions of Bouchard are amply confirmed.

It may help some of us to a more just appreciation of the etiological significance of autointoxication to recall that each of the mineral nutrients sodium, calcium, magnesium and potassium in the suitable dose, are distinctly poisonous and that of the group potassium is many times more toxic than any of the others, —and that there is a distinct consensus of opinion among our biological chemists, that we have yet much to learn as to the chemistry of autointoxication.

### THE PURINS AND HEREDITY.

The subject of heredity is without question one of the most interesting and most important which can engage the attention of medical men; in our work, our studies and in our hopes and imaginations we are constantly and incessantly confronting problems of heredity, and these heredity problems are frequently the most tragic of our personal or professional experiences.

Most unfortunately our problem,—the question of the heredi-tability of the purin diathesis,—leads us into the thick of a fight, the most strenuously contested question in all this literature,—the question of the hereditary transmission of acquired characters. Time will not permit me to present even an abstract of the arguments of the opposing sides of this question; it must suffice to mention the names of Galton, Mendel, Weismann, and Thompson as prominent in the opposition; of Lamark, Herbert Spencer, and Luther Burbank as of those in the affirmative. To my mind the work of Burbank has settled the question for all times. The seedless orange and the thornless cactus are surely instances of acquired characteristics, and Burbank has given us these in successive generations. The literature of this subject is most luminous and inspiring. One cannot read it without enlightenment upon a most vital matter; the questions of heredity and environment, of nature and nurture—these to medical men must ever be matters of the most supreme interest and importance.

My own conclusion is that the evolution the progress of man is mainly dependent upon the fact of the transmissibility of acquired characteristics; that as environment and nurture permit the more fortunate of men to acquire and to transmit desirable uplifting characteristics even so men less fortunate do acquire and do transmit diatheses, and of these the purin diathesis is easily acquired and securely transmitted to future generations.

### THE PURINS,—ENDOGENOUS, EXOGENOUS.

The work of comparative physiological chemistry should also be interesting to the medical man; of all men his work is biological, his facts are biological, and his hopes are biological, and biology is what comparative physiological chemistry can reveal to us of the nature of life in plants and animals. Students have long known that plants have the power to take from the air, the water, and the soil the elements oxygen, hydrogen, nitrogen, carbon and others and to combine these elements into the chemical substances, the proteids, the sugars, the starches, the oils; the tissues of the plants, their fruits and their seeds, many of them quite desirable for the use of man.

The forces which are seen in operation in these upbuildings of plant tissue are those of life, of the light and heat of the sun and of the chemical elements engaged; and the processes involved are known as those of photosynthesis, of chemosynthesis, and of photochemosynthesis. When I began the study of medicine and for many years thereafter students believed that a wide gulf separated the chemical processes and products of plants and animals; and one of the more prominent of the peculiar characteristics of plants was their monoply of the power of synthesis.

We were taught that plants build up, animals tear down; plants anabolise, animals catabolise; the relations of plants to oxygen was the only prominent exception to this rule; and this exception was always used as an illustration of the extreme diversity, the wide gulf separating the two kingdoms, plants and animals.

Some students now know that this apparent diversity of vital processes in plants and animals was only apparent by reason of our ignorance; it was never real. The unification of our knowledge of biology, the contribution of comparative physiological chemistry,—has been aided and advanced materially by the study of the endogenous and the exogenous purins, and here it will be well to add of plants and animals. Some of the more prominent facts of the metabolism of nitrogen closely related to the division of our subject now under consideration which are modern are:

1. That animals like plants have a distinct power of constructing proteid cells and tissues from elemental substances, and (2) that plants like animals are constantly catabolising their own proteid tissues; (3) that as urea is the catabolic product of proteid catabolism in man, ammonia is that of plants; (4) that as in man proteid catabolism is not always complete but frequently results from lack of thoroughness in suboxidation products of which the purin bodies are instances, even so in plants many such bodies are produced; and when purin bodies are so produced in plants they are known as endogenous purins. When in man by the operation of the chemosynthetic power of his tissues he builds purin bodies from a purin free diet; or when while fasting or when upon a diet free from purins he continues to excrete purin bodies, such are called endogenous purins.

Again, when man is fed generously upon a purin rich diet it is observed that the purin content of his excretions largely exceeds the normal quantity of endogenous purin, and this excess is called exogenous purin. We will see the practical bearing of these facts when we recall that in differing degrees the purin bodies are poisonous; that the excretion of the exogenous purins means work for heart, skin and kidneys; and that in the exigencies of life and with greater particularity with life characterised by overheating and drinking and underexercise, the excretory organs, skin and kidneys, are sometimes behind in their daily work, —that such slowness of metabolism means retention of exogenous purins,—and this means disease.

Our ability to prevent or cure such disease depends upon our competency in a given case to measure accurately the purin content of the daily diet, to estimate intelligently the rate of endogenous purin excretion for the individual, to measure accurately the rate of exogenous purin excretion, and then to estimate the fact and the rate of exogenous purin retention. Medical men are justly proud of the life-saving, health-giving achievements of modern scientific medicine. I know of none of wider applicability or greater potentiality than Emil Fischer's discovery of the purin nucleus, and the purins endogenous and exogenous.

### THE PURIN FOODS—RICH, POOR, AND FREE.

The writings of Emil Fischer and I. Walker Hall furnish the analyses of many of our more common foods and drinks, with the facts of their purin content or their freedom from the same. This knowledge is of inestimable value because of the enormous importance of the role of foods in man's environment—his nurture.

For centuries and centuries we have been groping and stumbling in the dim light of little knowledge with the absolute conviction of the masterful importance of our food supply, now with the firm belief that men were hurt by eating too much meat; again that men were injured by drinking too much beer and ale; still again that men were made ill by using too much tea, coffee and oatmeal. Out of these convictions and beliefs have arisen tribes and parties, sects and faddists without number; men having more faith than knowledge. In the great majority of cases the enthusiasm of the belief came from a personal observation of serious damage done by the use of a certain food and distinct betterment following the non use of that food.

Emil Fischer's contribution of the chemical composition of the purin nucleus making possible the detection of the purins whenever or wherever present, is bringing and will bring order to this chaos, the clear light of the sun where before was twilight or darkness, help to the afflicted, hope to those who are sore distressed. To my mind it is a fact of the first significance to find the purins widely distributed through our animal and vegetable foods and our more common drinks and beverages.

The following tables are from I. Walter Hall's book "The Purin Bodies of Food Stuffs."

*The Purin Rich Foods.*—Liver, kidneys, sweetbreads, beef, veal, pork, meat soups, salmon, halibut, chicken, tea, coffee, cocoa, beer, ale, porter, oatmeal, beans, peas, lentils, asparagus.

*The Puirn Poor Foods.*—Codfish, plaice, mutton, pork-fat, potatoes, onions, chocolate.

*The Purin Free Foods.*—Milk, cream, butter, cheese, eggs, mutton fat, veal fat, rice, wheat flour, white bread, cabbage, lettuce, fruits.

Thanks to our more accurate knowledge of metabolism and nutrition we are now in position to give advice as to the proper use of these several foods. There is a consensus of both the art and the science of medicine, of experience and knowledge in the advice that the purin rich foods are to be recommended for all children, youths and those in early manhood, save when contraindicated by the facts of heredity; for hard workers in the open air; for nursing mothers and for those convalescing from exhausting fevers; for cases of anemia, chlorosis and tuberculosis.

The use of purin rich foods should be carefully regulated in cases of purin diatheses or purin diseases; gout, acute, sub-acute, or chronic; rheumatism, acute, sub-acute or chronic; and functional disturbances or complaints where there is urinary evidence of perverted purin metabolism; all cases of premature senescence; all men past three score years of age; cases of persistent high arterial tension, of suspected arteriosclerosis, many cases of feeble liver, kidney and heart; all cases where occupation exposes to bad air, as Buffalo school teachers, clerks and stenographers.

### THE PURINS AND OLD AGE.

The work of the medical man is one of unusual opportunity and singular responsibility. The popular medical specialty of the near future will be—keeping your patients young. Our success in this specialty will be in proportion to our devotion to the important truths of heredity and environment, of nature and nurture. We may well take for our motto the classic saying: "A man is no younger than his arteries." We must prevent senescence, premature old age, and arteriosclerosis, now all too frequent in the fifth, sixth and seventh decades.

We know that intelligent nutrition in mid-life from fifty to seventy requires much less nitrogen than was assumed to be requisite fifty years ago. We should know that purin toxemia, purin retention, makes a man old before his time. Bulk for bulk the oxidation of purins in man is done by kidneys, liver and muscles, which means that effectively it is done by muscles, liver and kidneys. We will do well to declare by precept and example our confidence in moderation in eating and drinking—regular habits of vigorous exercise to keep the skin and muscles young—that these may help to keep the liver, kidneys and arteries from senescence.

### MODERN DIETOTHERAPY.

Dietotherapy today rests upon a broad, wide and deep foundation,—a firm foundation. The recent advances in the accurate knowledge of the chemical composition of foods; the pathologic metabolic consequences of overuse and underuse of foods; the why of the evil effects of certain foods in the production of disease; the enormously beneficial results of fasting and dieting; of moderation in eating in the presence of certain diatheses or diseases; our knowledge of the functions of the internal secretions; of the processes of nutrition, of constructive and destructive metabolism; of the role of the different microörganisms and ferments of the stomach and intestines; of urinology and the evidence the urine furnishes as to the errors of metabolism; the facts of high and low blood pressures and the relation of such to various dietotoxins,—the rapidly accumulating evidence of biological chemistry,—makes dietotherapy today of enormous significance and importance, the much neglected and the most hopeful field of medical practice.

CONCLUSIONS.

In conclusion we may note:

1. The significant hopefulness and inspiration of our practical biochemical knowledge of the purins of plants and animals.

2. The enormous responsibility and obligation involved in the care of cases in which habits of eating and drinking may so impress the body of the individual as to produce defective characteristics capable of transmission to posterity. Also the peculiar responsibility of the cases of diathesis and of hereditary diseases.

3. The opportunity for more accurate study of cases of defective metabolism given by our recent knowledge of endogenous and exogenous purins.

4. The possibility of more practical intelligence in prescribing a suitable diet in a given case from our knowledge of an enlarged and varied number of purin rich, poor and free foods.

5. The economical, sociological, and professional importance of the fact, that man's effective ability for performance, for enjoyment, for example, is often reached at the fifth decade and too often lost in the sixth, when it should be prolonged to the ninth.

433 Franklin Street.

